# Trade agreements and tobacco control policy: analysis of the impact of FCTC on regulatory contents of trade agreements from 2001 to 2019

**DOI:** 10.1186/s12992-023-00979-w

**Published:** 2023-10-20

**Authors:** Tzu-Ying Chen, Ying-Jun Lin, Tung-Liang Chiang, Feng-Jen Tsai

**Affiliations:** 1 Institute of Health Policy and Management, National Taiwan University, Taipei City, Taiwan; 2 School of Law - Financial and Economic Law, Chung Yuan Christian University, Taoyuan City, Taiwan; 3 Ph. D. Program and Master Program in Global Health and Health Security, College of Public Health, Taipei Medical University, No.301, Yuantong Rd, New Taipei City, Taiwan

**Keywords:** Tobacco, WHO Framework Convention on Tobacco Control (FCTC), Trade agreement, Trade measures

## Abstract

**Background:**

This study aims to analyze the impact of Framework of Convention of Tobacco Control (FCTC) on regulatory contents of trade agreements from 2001 to 2019.

**Methods:**

A search of trade agreements from‘WTO Regional Free Trade Agreement Database’ using keywords including “tobacco”, “cigarette”, “smoking” and “FCTC” from May to August 2020 resulted in a total sample of 268 trade agreements, from which 69 trade agreements were coded and analyzed. Provisions in trade agreements, identified via the aforementioned keywords, were categorized into 6 trade measures. The word counts of the provisions containing; FCTC; were calculated. Chi-square tests were applied to analyze the differences of regulatory patterns between different time frames. The import and export values (USD) of tobacco products under trade agreements containing the term “FCTC” were further collected from the “International Trade Statistics 2001–2020” for understanding the impact of the provision on the trade flow.

**Results:**

Among 69 agreements, the percentage of trade agreements containing keyword as “FCTC” increased significantly from 0% to 2011 to 12% after 2011. A significant decrease of using trade measures as “the exclusion list” was found after 2011 (from 10% to 0). The word counts of provisions containing; FCTC; increased from 24 words in 2011 to 164 words in 2018, and the content of the provisions became more concrete over time. There are six trade agreements containing “FCTC”, and all these 6 agreements were ratified by European Union (EU). Despite EU ratified trade agreements with “FCTC”, the import and export values of tobacco products between EU and the other party countries increased with time. But the gap of average trade values between trade agreements with and without “FCTC” being widened with time.

**Conclusions:**

As a first study evaluated the impact of FCTC on regulatory contents of trade agreements, our study results showed that after countries signed trade agreements containing keyword FCTC, the regulatory contents changed significantly. Further studies are recommended to understand the reason and criteria for incorporating FCTC provisions into trade agreements, especially in the EU.

## Background

Tobacco smoking causes approximately 8 million deaths worldwide every year [1, 2]. Smoking also leads to diseases such as cancer, heart disease, stroke, and lung diseases among smokers and non-smokers [3–5]. In response, the “WHO Framework Convention on Tobacco Control (FCTC)” was adopted by the World Health Assembly (WHA) on May 21, 2003 to “protect present and future generations from the devastating health, social, environmental and economic consequences of tobacco consumption and exposure to tobacco smoke” [6].

FCTC has contributed to significant and rapid progress in implementing national tobacco control measures like regulating smoke-free public places and health warnings on cigarette package in many countries [7, 8]. The rates of adopting national tobacco control measures increased around 2 to 4 times from 1999 to 2019 [9]. Furthermore, following FCTC ratification, the global prevalence of tobacco smoking decreased from 24.3% to 2005 to 18.7% in 2020, some of which may be attributable to FCTC adoption [10]. However, despite the success of FCTC on promoting national tobacco control policies, it has been criticized for being slow and insufficient in regulating the international trade of tobacco products [7, 11].

The World Trade Organization (WTO) plays a key role in regulating the international trade of tobacco products. As an organization aims to promote trade liberalization by reducing and eliminating tariffs and other trade barriers [12], WTO encourages members to negotiate new trade regulations to expand market access [13]. Tobacco products have also been part of the liberalization of the trade in goods. However, several studies have pointed out the possible negative impact of trade on health through increasing accessibility of unhealthy products, including tobacco products [14–16]. The negative impact of trade on tobacco control may be evidenced by the parallel growth between trade agreements and cigarette consumption. Previous studies found that the number of trade agreements enforced increased sharply worldwide from 12 to 1980 to 284 in 2016 [16, 17]; meanwhile, global annual cigarette consumption also rose from 4.3 trillion in 1980 to 5.7 trillion sticks in 2016 [18].

The FCTC noticed the possible harmful impact trade liberalization may have on tobacco control and public health. Mamudu et al. (2010)’s important research indicated that the lack of consensus between trade and health caused the absence of explicit FCTC trade provision. He also argued that the public health community should become more involved in trade and health issues at all levels of governance and press the FCTC Conference of the Parties (COP) for clarification of this critical issue [19]. In addition to the measures recommended to reduce the supply of tobacco products, the FCTC also recognizes the role of international agreements, either bilateral or multilateral, in maintaining the consistency of global governance of tobacco control [20, 21]. Given the purpose of regulating cross-border trade in goods, trade agreements are part of the international agreements highlighted by the FCTC. In theory, trade agreements could positively contribute to tobacco control by approaches, like excluding tobacco from the commitments of trade liberalization and securing the compatibility of trade measures with the FCTC provisions [20]. However, the effect of trade agreements on maintaining the international governance of tobacco control is questionable [14]. The concern of normative conflict between trade and public health has been reflected also in decisions adopted by the Parties of the FCTC. Specifically, the decision of the Fourth Conference of the Parties (COP4) in 2010 underscored the FCTC’s position on reconciling trade agreements and tobacco control policies [22]. COP5 and COP6 further discussed the guiding principle to counterbalance the negative impact of trade on health including duty-free tobacco [23, 24]. One of the major recommendations that came out of COP5 and COP6included recommendations to consider prohibiting the importation of tobacco products by international travelers and to restrict the sale of tax-free or duty-free tobacco products.

Previous studies pointed out that the negotiation process before FCTC ratification can accelerate the pace of regulation in countries [25, 26]. Therefore, theoretically speaking, although FCTC COP recommendations and decisions are not legally binding, these documents may still have the impact on promoting government’s policies for trade and health consistency. In addition, based on the rationale that the inclusion of FCTC provision in the trade agreement represented the awareness and willingness of the countries to put efforts in tobacco control, the trade flow on tobacco of countries who signed the trade agreements should be reduced. Based on such hypothesis, we conduct this study to identify if the recommendations and decisions of FCTC COPs have impact on regulatory contents in trade agreements. Further, if regulatory contents changes were found, the changes of trade flows were further determined.

To our knowledge, there has not yet been a study evaluating the impact the FCTC has had on trade agreements. For understanding the issue, the objectives of this study are: [1] Compare the regulatory contents of trade measures concerning tobacco control in trade agreements between different time frames; [2] Analyze the changes in the trade agreements containing; FCTC; with time; and [3] Analyze the trends of import and export values of tobacco products under trade agreements enforced from 2001 to 2019.

## Methods

### Data collection

We collected all the trade agreements with a date of entry into force between 1 and 1995 to 31 July 2020 in the “WTO Regional Free Trade Agreement Database (FTA/RTA Database)” from May to August 2020. Among these 25 years, there were 268 trade agreements in total. And all these 268 trade agreements were collected for further analysis.

Then trade agreements that were not written in English and did not contain the keywords “tobacco”, “cigarette”, “smoking”, or “Framework Convention of Tobacco Control (FCTC)” were excluded. And irrelevant trade agreements, referred to trade agreements that did not substantially address tobacco control were further excluded after content review. For example, ‘tobacco’ only present in the title ‘Section IV Prepared foodstuffs; beverages, spirits and vinegar; tobacco and manufactured tobacco substitutes’ in the annex of the “Canada – Israel Trade Agreement”. With the fact that the keyword only presented as an item on the general list without further impact on regulatory content, it is considered as irrelevant trade agreement in our study. Two researchers independently screened all the trade agreements to exclude irrelevant ones. The inter agreement rate was 100%.

**Fig. 1:**
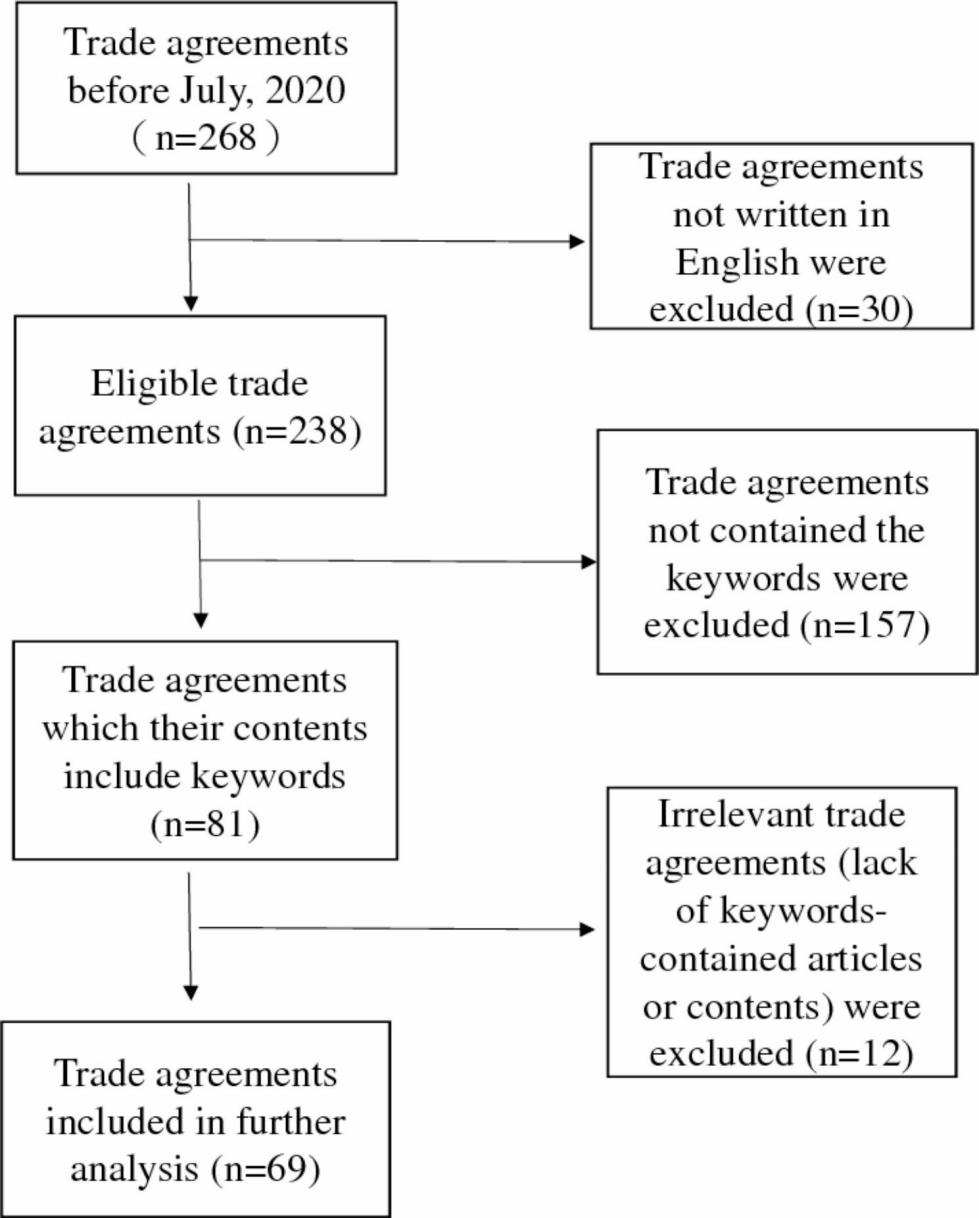
Data collection process by PRISMA flowchart

The process was performed following the Preferred Reporting Items for Systematic Reviews and Meta-Analyses (PRISMA) [27]. The selection process resulted in 69 trade agreements that were included for further analysis (Fig. 1).

### Characteristics of trade agreements and trade measures

Information collected from the trade agreements included the date of entry into force, which countries were party to the treaty, and the text sections containing the keywords.

The keywords were shown in the provisions of 69 trade agreements. The provisions containing keywords were collected and categorized into six trade measures, including “tariffs”, “the exclusion list”, “rules of origin”, “quantitative restrictions”, “non-tariff measures”, and “other exclusion measures” (see supplements). Based on the reason that these six trade measures are the most common measures used in trade agreements for explaining trade barriers between contracted countries, we used these six trade measures to classify the measures used in the trade agreements [28]. Tariffs are duties imposed on imported goods. Tariffs are the most common barrier for goods to access into the market [29]. “The exclusion list” is also a popular way for countries to exclude certain harmful products from their market-opening commitments. If the goods were excluded, governments would not need to ensure that health measures are consistent with trade rules and tobacco companies could not sue over government control policies that contravene investment guarantees [30, 31]. “Rules of origin” are used to prevent the import of any particular commodity from entering through the country with the lowest duty on the item in question and being re-exported to other countries in trade agreements [32]. “Quantitative restrictions” are trade measures that control importation or exportation by fixing the volume or value of products [29, 33]. “Non-tariff measures” refer to all other governmental interventions that affect the cross-border flow of commodities [29].

In addition, there are other measures that have not traditionally been included trade agreements or considered in the literature that could be used in trade agreements for tobacco control, such as restrictions on duty-free entry, distribution wholesale, and retail sale of tobacco products. The purpose of these trade measures is to exclude tobacco products from easy access to the market; we refer to these measures as “other exclusion measures”.

### Trends of import and export values of tobacco products

Among 69 trade agreements, there are 6 agreements containing the keyword as “FCTC”. All these 6 agreements were ratified by European Union (EU). With the finding, we additionally collected import and export values (USD) of tobacco products between EU and party countries from 2001 to 2019 from the WTO database under agreements for further analysis.

For the consistency of trade data, tobacco and manufactured tobacco substitutes were classified as Item 24 under the Harmonized System Code (HS Code). Hence, we collected Item 24 data from the database of “International Trade Statistics 2001–2020” in the “International Trade Centre” (ITC) website from May to August 2020. The yearly trade flow data available in ITC is mainly based on “UN Comtrade Database” and covers more than 90% of world trade data [34].

### Analysis

To capture the influence of the FCTC on trade agreements, the time scale of analyzed trade agreements were divided into three phases by two crucial events: the date of the FCTC coming into force (27 February 2005) and the ratification date of the first trade agreement containing provisions from the FCTC (1 July 2011).

Word counts were calculated and content analysis was conducted to compare regulatory contents included in trade agreements before and after FCTC ratification.

Comparing trade agreements negotiated before and after 2005 captures the influence of the FCTC on trade agreements. Comparing trade agreements enforced before and after 2011 allows us to examine if there is a mutual learning effect (or imitation effect) between trade agreements. The two sets of comparisons reveal changes in normative contents of trade agreements concerning the 6 trade measures and FCTC contents.

Chi-square test was used to analyze differences between categorical variables. In this study, chi-square test was used to compare the differences in trade measures used in agreements between different time frames. In detail, chi-square test was used to compare the regulatory content of trade agreements that coded into the six categories between pre-2005 and post-2005 agreements. Also, chi-square test was used to compare the regulatory content of trade agreements between pre-2011 and post-2011 agreements. Import and export values (USD) of tobacco products in countries party to the included trade agreements were calculated. The statistical analysis was performed using IBM SPSS Statistics 20.

## Results

Among the 69 trade agreements included in this study, there were 19 trade agreements that came into force before 2005 (27.5%), 20 trade agreements that came into force between 2005 and 2011 (28.89%), and 30 trade agreements that entered into force after 2011 (43.47%).

Additionally, 99 countries entered into 69 trade agreements. Among them, 91 countries (91.92%) are party to the FCTC; 84 countries are WTO members and 6 countries are WTO Observers (90.91%). 85 countries (85.86%) are both party to the FCTC and WTO member states or observers.

### Comparison of trade measures in trade agreements between different time frames by chi-square test

Comparisons of trade measures in trade agreements between different time frames by chi-square test are shown in Table 1. The difference in the types of trade measures in trade agreements before and after the FCTC came into force in 2005 are shown in Table 1 A. The percentage of measures as “tariffs”, “non-tariff measures”, “other exclusive measures” and “FCTC contained contents” is higher in trade agreements negotiated after 2005 than these negotiated before 2005. But the difference is not statistically significant.

**Table 1A:** Inclusion of keywords in trade agreements enforced before and after 2005

Trade measures in trade agreements	Total	Agreements before 2005/2/27 (n = 19)		Agreements after 2005/2/28 (n = 50)		Chi-square
	n	n	%	n	%	
**Tariff**						
Yes	27	6	31.6	21	42	0.62
No	42	13	68.4	29	58	
**The exclusion list**						
Yes	5	2	10.5	3	6	0.42
No	64	17	89.5	47	94	
**Rules of origin**						
Yes	36	13	68.4	23	46	2.77
No	33	6	31.6	27	54	
**Quantitative Restrictions**						
Yes	14	4	21.1	10	20	0.01
No	55	15	78.9	40	80	
**Non-tariff measures**						
Yes	11	1	5.3	10	20	2.23
No	58	18	94.7	40	80	
**Other exclusive measures**						
Yes	12	3	15.8	9	18	0.05
No	57	16	84.2	41	82	
**FCTC contained contents**						
Yes	6	0	0	6	12	2.50
No	63	19	100	44	88	

p < 0.05*

The differences in the types of trade measures before and after 2011 were showed in Table 1B. The percentage of trade agreements using trade measure as “the exclusion list” in trade agreements enforced before 2011 (10.5%) was significantly higher than agreements enforced after 2011 (0%) (p < 0.05). The percentage of trade agreements using FCTC after 2011 (20%) was significantly higher than agreements negotiated before 2011 (0%) (p < 0.05).

**Table 1B:** Inclusion of keywords in trade agreements enforced before and after 2011

Trade measures in trade agreements	Total	Agreements before 2011/6/30 (n = 39)		Agreements after 2011/7/1 (n = 30)		Chi-square		
	n	n	%	n	%			
**Tariff**								
Yes	27	14	31.6	13	43.3	0.39		
No	42	25	68.4	17	56.7			
**The exclusion list**								
Yes	5	5	10.5	0	0	4.15*		
No	64	34	89.5	30	100			
**Rules of origin**								
Yes	36	22	68.4	14	46.7	0.65		
No	33	17	31.6	16	53.3			
**Quantitative Restrictions**								
Yes	14	7	21.1	7	23.3	0.30		
No	55	32	78.9	23	76.7			
**Non-tariff measures**								
Yes	11	4	5.3	7	23.3	2.16		
No	58	35	94.7	23	76.7			
**Other exclusive measures**								
Yes	12	7	15.8	5	16.7	0.02		
No	57	32	84.2	25	83.3			
**FCTC contained contents**								
Yes	6	0	0	6	20	8.54*		
No	63	39	100	24	80			

p < 0.05*

### The analysis of FCTC contents in trade agreements

The text of provisions that were taken directly from the FCTC in the six trade agreements included in this study are shown in Table 2. Five of the trade agreements (83.34%) incorporated the FCTC provisions in chapters related to of Public Health while 3 agreements (50%) included the FCTC provisions in the chapters related to taxes. In addition, there are 2 agreements (33.33%) that had FCTC provisions in both public health and tax chapters.

**Table 2:** Details of the trade agreements with FCTC-contained contents

Contacted countries	Year of Entry into Force	Contents of the 6 FCTC-contained trade agreements	
		Chapter of public health related title	Chapter of taxation
EU - Republic of Korea^1^	2011	Article 21: The Parties shall endeavor to promote implementation of international health agreements such as the International Health Regulations and the Framework Convention on Tobacco Control.	N/A
EU - Central America^2^	2013	Article 44: Cooperation may further encourage the development, implementation and promotion of international health law, including the International Health Regulations and the World Health Organization Framework Convention on Tobacco Control.	N/A
EU – Ukraine^3^	2014	N/A	Article 352: The Parties shall develop their cooperation and harmonise policies in counteracting and fighting fraud and smuggling of excisable products. This cooperation will include, inter alia, the gradual approximation of excise rates on tobacco products, as far as possible, taking into account the constraints of the regional context, including through a dialogue at regional level and in line with the World Health Organisation Framework Convention on Tobacco Control of 2003. To this end, the Parties will look to strengthen their cooperation within the regional context.
EU - Republic of Moldova^4^	2014	Article 113–114(Public Health): The Parties agree to develop their cooperation in the field of public health, with a view to raising the level of public health safety and protection of human health as a precondition for sustainable development and economic growth. The cooperation shall cover, in particular, the following areas: (1) prevention and control of non-communicable diseases, mainly through exchange of information and best practices, promoting healthy lifestyles and addressing major health determinants, such as nutrition and addiction to alcohol, drugs and tobacco; (2) full and timely implementation of international health agreements, in particular the International Health Regulations and the World Health Organisation Framework Convention on Tobacco Control of 2003.	Article 55: The Parties shall develop their cooperation and harmonise policies in counteracting and fighting fraud and the smuggling of excisable products. That cooperation will include, inter alia, the gradual approximation of excise rates on tobacco products, as far as possible, taking into account the constraints of the regional context, including through a dialogue at regional level and in line with the World Health Organisation Framework Convention on Tobacco Control of 2003 (WHO FCTC). To that end, the Parties will strive to strengthen their cooperation within the regional context.
EU – Georgia^5^	2014	Article 355–356: The Parties agree to develop their cooperation in the field of public health, with a view to raising the level of public health safety and protection of human health as an essential component for sustainable development and economic growth. (1) prevention and control of non-communicable diseases, mainly through exchange of information and best practices, promoting healthy lifestyles, physical activity and addressing major health determinants, such as nutrition, addiction to alcohol, drugs and tobacco; (2) effective implementation of international health agreements to which the Parties are party, in particular the International Health Regulations and the Framework Convention on Tobacco Control.	Article 283: The Parties shall develop their cooperation and harmonise policies in counteracting and fighting fraud and smuggling of excisable products. This cooperation will include, inter alia, the gradual approximation of excise rates on tobacco products, as far as possible, taking into account the constraints of the regional context, and in line with the World Health Organisation Framework Convention on Tobacco Control. To that end, the Parties will look to strengthen their cooperation within the regional context.
EU – Armenia^6^	2018	Article 28: The Parties shall develop their cooperation with a view to reaching shared policies for counteracting and fighting fraud and the smuggling of excisable products. The cooperation shall involve the exchange of information. To that end, the Parties shall look to strengthen their cooperation within the regional context and in line with the World Health Organization Framework Convention on Tobacco Control of 2003. Article 91–92(Cooperation in the area of health): (1) The Parties shall develop their cooperation in the field of public health with a view to raising its level, in line with common health values and principles, and as a precondition for sustainable development and economic growth. (2) Cooperation shall address the prevention and control of communicable and non-communicable diseases, including through the exchange of health information, the promotion of a health-in-all-policies approach, cooperation with international organisations, in particular the World Health Organization, and the promotion of the implementation of international health agreements such as the World Health Organization Framework Convention on Tobacco Control of 2003 and the International Health Regulations.	N/A

^1^ EU - Republic of Korea Trade agreement, Article 21(3), p.34.

^2^ EU - Central America Trade agreement, Article 44(4), p.17.

^3^ EU – Ukraine Trade agreement, Article 352, p.141; Article 427(1c), 428, p.158.

^4^ EU - Republic of Moldova Trade agreement, Article 55, p.20; Article 114(c, f), p.32.

^5^ EU – Georgia Trade agreement, Article 283, p.108; Article 356(c, f), p.121.

^6^ EU – Armenia Trade agreement, Article 28, p.15; Article 92, p.29.

### Word counts analysis of FCTC contents in trade agreements

**Fig. 2:**
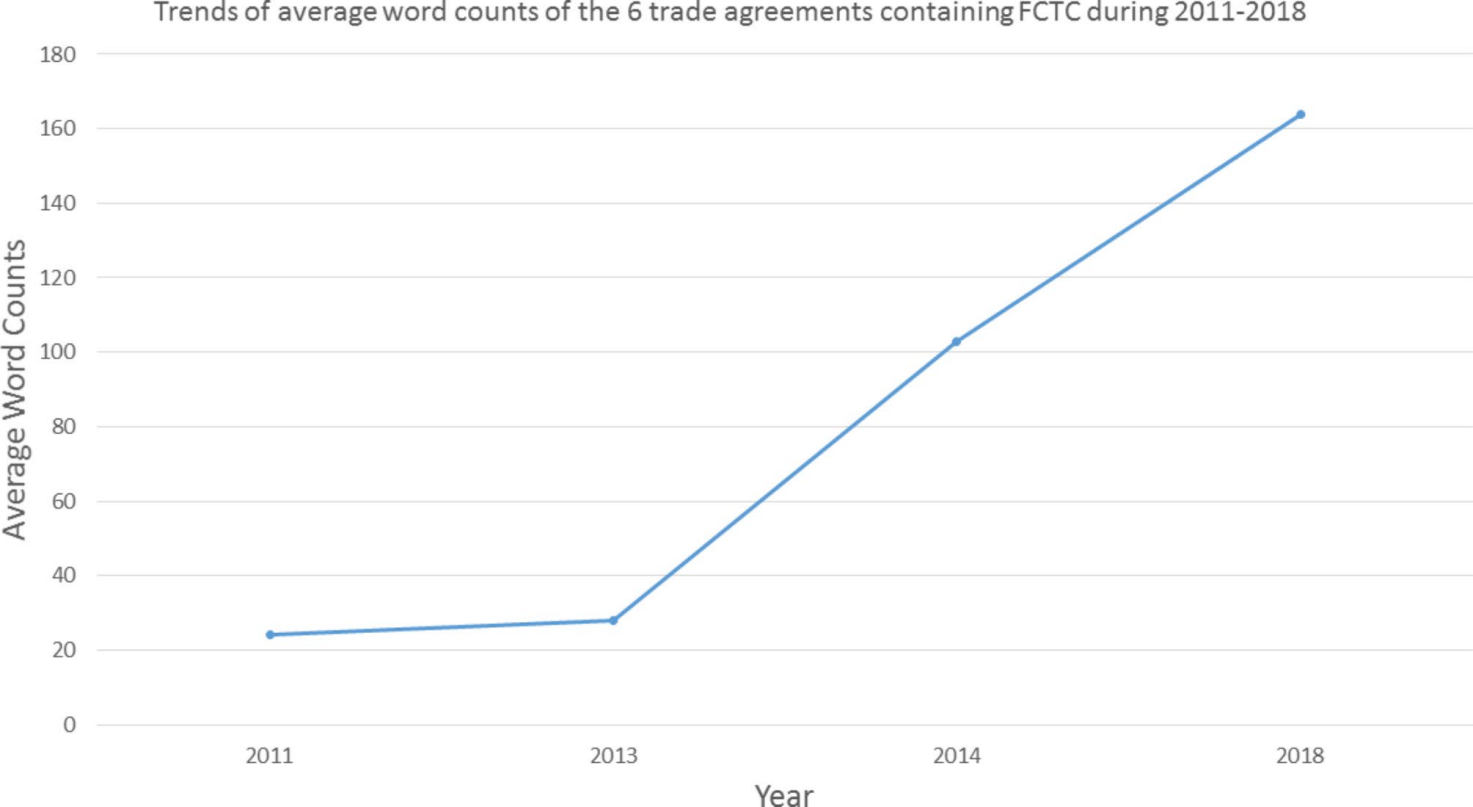
Trends of average word counts of the 6 trade agreements containing FCTC during 2011–2018

The word counts of provisions taken directly from the FCTC increased with time. For the articles listed in a public health related chapter, there were 24 words regarding FCTC in the trade agreements ratified in 2011 and 28 words in agreements enforced in 2013. The trade agreements ratified in 2018 included 164 FCTC related words, five more times than before (Fig. 2). In tax related chapters, there were 82 words regarding the FCTC on average in the 3 trade agreements ratified in 2014.

### Content analysis of FCTC contents in trade agreements

In Table 2, articles listed in public health chapters often mentioned the FCTC in conjunction with International Health Regulations (IHRs) [35] to promote the implementation of international health agreements. This kind of regulatory pattern appeared in trade agreements ratified in 2011 and 2013. The trade agreements ratified in 2014 went further to give normative details. The trade agreement requires the contracting Parties to implement the international health law by specific actions. The actions included raising the level of public health safety, protecting human health as an essential component for sustainable development and economic growth, and preventing and controlling of non-communicable diseases through information exchange and best practices. These required actions are supposed of favoring effective implementation of IHRs and the FCTC.

The trade agreement ratified in 2018 not only repeated specific FCTC provisions used in the 2014 ratified agreements, but also incorporated a health-in-all-policies approach to promote cooperation among countries party to the treaty.

Three trade agreements ratified in 2014 encouraged the countries party to the treaty to introduce domestic restriction measures on tobacco products such as regulating exercise rates. These trade agreements also facilitated the implementation of the FCTC by addressing the importance of combating the smuggling of tobacco products. Likewise, the 2018 ratified agreement also stated the importance of combatting the smuggling of excisable products. The difference is that the 2018 ratified trade agreement addressed illegal products in the public health chapter instead of the taxation chapter.

### Trends of import and export values of tobacco products under trade agreements

**Fig. 3:**
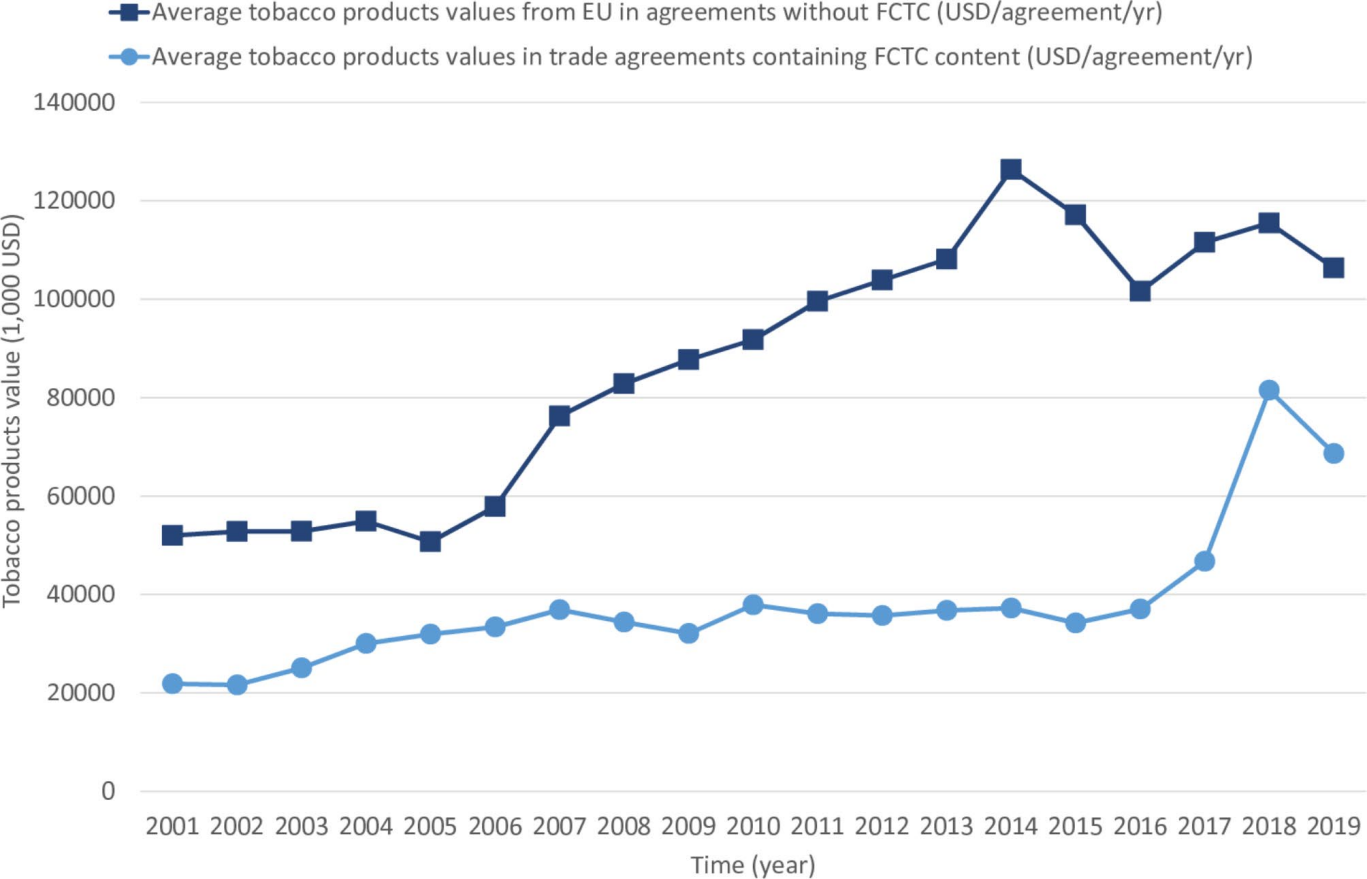
Trends of the import and export values of tobacco products under trade agreements led by the EU from 2001 to 2019

Trends of import and export values of tobacco products under trade agreements led by the EU from 2001 to 2019 are shown in Fig. 3. The average import and export values of tobacco products under the trade agreements with FCTC contents grew slowly from USD 21,856,000 in 2001 to USD 37,015,000 in 2016. After 2017, the import and export values of tobacco products increased sharply and reached a peak in 2018 (USD 81,512,000). Simultaneously, the average import and export values of tobacco products in the EU-led trade agreements that didn’t contain FCTC content increased sharply from USD 50,696,000 in 2005 to USD 126,359,000 in 2014, then slightly reduced after.

Additionally, the average trade values of tobacco products of the countries involved in the 21 EU-led trade agreements without FCTC contents is higher than the average trade values of the countries involved in the 6 EU-led trade agreement with FCTC content. The gap in the average import and export values between the two groups widened from USD 3,666,000 in 2005 to USD 89,130,500 in 2014. The gap increased more than 24 times.

**Fig. 4:**
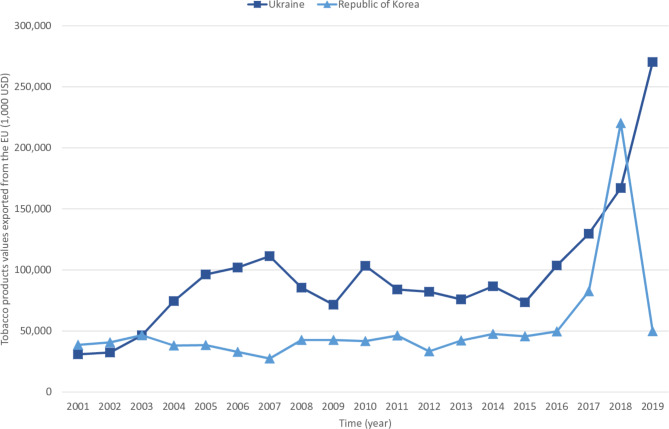
The trends of export values of tobacco products from EU to Ukraine and Republic of Korea during 2001 to 2019

Among countries that signed trade agreements with the EU that contained FCTC content, Ukraine and Korea were the top 2 countries with the highest trade flows of tobacco products with EU. The average export values from EU to Ukraine and Korea are USD 182,637,000 and USD 100,619,000 per year as shown in Fig. 4. Although Ukraine and Korearatified their trade agreements with the EU in 2011 and 2014, the trade flow of tobacco products between EU and the two countries still increased after ratification. The export values of tobacco products from EU to these two countries accounted for more than 90% of their import and export values of tobacco products. The value of tobacco exports grew more rapidly after 2016, and reached a peak in 2018 (USD 387,398,000).

## Discussion

As a first study evaluated the impact of FCTC on regulatory contents in trade agreements, we found that FCTC do have the impact on regulatory contents of trade agreements overtime. Also, the trade flows change when regulatory contents change were found.

In specific, the use of trade measure “the exclusion list” in trade agreements significantly decreased from 10% to 2011 to 0% in 2011. With significant increase of “FCTC contained contents” in trade agreements after 2011, the word counts of FCTC contents also increased with time. In addition, we found only EU-led trade agreements contain the term “FCTC”. With the finding, an additional analysis of trade values showed that, although the EU signed trade agreements with “FCTC”, the trade values of tobacco products still increased with time. But the gap in the average import and export values between countries involved in trade agreements with and without the FCTC content being widened with time.

Our results show that the use of “the exclusion list” in trade agreements decreased significantly. This trend might reflect the increased focus of global community on the issue of international trade and tobacco control [19]. It also reflects changing ideas about the use of exclusion list for tobacco products [4]. Prior to 2011, governments preferred approaches such as “the exclusion list” to avoid inconsistency between health measures and trade rules and because there were concerns that tobacco companies would sue [30]. However, McGrady (2007) argued that exclusion lists are problematic, especially because a contradiction between increasing and decreasing the price of tobacco and its relevant products might happen if the government failed consider the issue of domestic support. The exclusion policy would indirectly support protectionism and may thereby undermine the potential benefits of trade liberalization [36]. These reasons may explain the reduction in the use of exclusion lists to control tobacco products.

The finding that all the six trade agreements containing FCTC provisions were ratified after 2011 might be explained by the development of the COP4 of the FCTC in 2010 [22, 37]. In addition, one of the outcomes of COP4 [37] was a request that the FCTC to cooperate with the WTO, which may explain increased FCTC adoption. Since the cooperation between the WHO and the WTO signals a supportive relationship instead of parallel issues between trade and public health policies. FCTC contents were appeared in trade agreements and turned into more concrete with increased word counts after.

Similarly, the adoption of the “Protocol to Eliminate Illicit Trade in Tobacco Products” in FCTC COP5 may be the reason for the appearance of content related to the smuggling of excisable products. The geographic connection might also be the reason for the mention of smuggling in the agreements.

Interestingly, we found the six trade agreements containing FCTC provisions were all signed by the EU. The involvement of the EU implies that the EU played an active role in supporting the implementation of the FCTC [38]. The tobacco industry even tried to intervene the process of legislating the EU Tobacco Products Directive (hereinafter “TPD”) in 2001. Despite remaining political pressure and tobacco industry influence of campaigns to amend the TPD, the EU insisted on including FCTC Article 5.3 in the TPD Revision legislation in 2014. This article is not only a tool against industry interference but also keeps policymakers accountable and transparency when dealing with the tobacco industry [39]. After the TPD Revision, the EU continued to promote tobacco control policies vigorously in the FCTC and further incorporated FCTC provisions in new trade agreements.

The findings that the average import and export value of tobacco products of the six EU-led trade agreements containing FCTC is lower might be the evidence that incorporating FCTC provisions in trade agreements might reduce the trade flow of tobacco products between countries. However, the increasing exportation of tobacco products from EU to Ukraine and Korea under trade agreements with FCTC contents raise uncertainties.

The finding showed that rhetorical commitments to FCTC implementation are not necessarily linked to a commitment to implement its provisions. In addition, the formal inclusion of text on FCTC implementation may be a necessary, but not sufficient, condition for reducing the value of tobacco trade. Therefore, further research is required to fully understand the connections between the inclusion of FCTC provisions in trade agreements and reductions in the availability of tobacco products.

Besides, the WTO RTA Database may not cover all the trade agreements in the world, however, the trade agreements collected from the WTO RTA Database are representative. Because the WTO members and observers already cover 164 economies which representing 96.4% of global trade value. Additionally, the transparency mechanism established by the WTO General Council in 2006, has compelled WTO members and observers to notify the WTO of any new trade agreement. This mechanism enhances the reliability and sensitivity of the data from the WTO RTA Database [40].

### Limitations

In this study, the Database of International Trade Statistics 2001–2019 of ITC comprises more complete information in the “value” but not the “quantity” of the export and import of tobacco products. Therefore, the trade performance of tobacco products does not indicate the quantity of tobacco products.

With the fact that keywords we used for searching as “tobacco” or “cigarette” were not showed in the “health exception provisions” in trade agreements, and the contents of such provisions were not analyzed in our study. Health exception provision might have potential contribution on tobacco control, further studies on the issue are recommended. In addition, a trade agreement which would encompass tobacco products without any keywords being used in this study is not covered by our study.

Similarly, the “Trans-Pacific Partnership (TPP)” shown in our study as regular agreement with keywords in its provisions of “Rules of Origin (ROO)” and “Other exclusive measures”. Since the TPP has provided a new possibility as the first regional trade agreement including tobacco carve-out measure by containing related language in the “general exceptions” chapter, further study on the issue is recommended.

Given that the EU is the exception in terms of trade agreements making explicit reference to FCTC and its implementation, the finding of this study is limited.

## Conclusions

As a first study evaluated the impact of the FCTC on trade agreements, we found that the FCTC first appeared in trade agreements in 2011. The usage of trade measures as “the exclusion list” for tobacco products in trade agreements significantly decreased after 2011. In addition, the EU is involved in all the trade agreements containing FCTC. Although the EU takes the lead in including FCTC contents in trade agreements, the flow of tobacco trade between the EU and its countries remains on an upward trend.

Our study results showed the possible impact of FCTC on trade agreements and trade flow on tobacco products. Further studies are recommended to better understand how FCTC contents are incorporated into trade agreements, especially in the EU.

## Data Availability

All the data in this research were obtained from publicly available sources.

## Abbreviations

FCTC: Framework of Convention of Tobacco Control
ITC: International Trade Centre
WHA: World Health Assembly
WTO: World Trade Organization
COP: Conference of the Parties
FTA: Free Trade Agreement
RTA: Regional Trade Agreement
PRISMA: Preferred Reporting Items for Systematic Reviews and Meta-Analyses
HS Code: Harmonized System Code
IHRs: International Health Regulations
TPP: Trans-Pacific Partnership
ROO: Rules of Origin
